# A Population-Based Intervention for the Prevention of Falls and Fractures in Home Dwelling People 65 Years and Older in South Germany: Protocol

**DOI:** 10.2196/resprot.3126

**Published:** 2014-03-31

**Authors:** Diana Klein, Kilian Rapp, Michaela Küpper, Clemens Becker, Torben Fischer, Gisela Büchele, Petra Benzinger

**Affiliations:** ^1^Department of Clinical GerontologyRobert-Bosch-HospitalStuttgartGermany; ^2^Institute of Epidemiology and Medical BiometryUlm UniversityUlmGermany

**Keywords:** falls, accidental falls, population-based intervention, fall prevention, community, aged, risk factors

## Abstract

**Background:**

Falls and fall-related injuries pose a major threat to older peoples’ health, and are associated with increased morbidity and mortality. In the course of demographic changes, development and implementation of fall prevention strategies have been recognized as an urgent public health challenge. Various risk factors for falls and a number of effective interventions have been recognized. A substantial proportion of falls occur for people who are neither frail nor at high risk. Therefore, population-based approaches reaching the entire older population are needed.

**Objective:**

The objective of the study presented is the development, implementation, and evaluation of a population-based intervention for the prevention of falls and fall-related injuries in a medium sized city in Germany.

**Methods:**

The study is designed as a population-based approach. The intervention community is a mid sized city named Reutlingen in southern Germany with a population of 112,700 people. All community dwelling inhabitants 65 years and older are addressed. There are two main measures that are defined: (1) increase of overall physical activity, and (2) reduction of modifiable risk factors for falls such as deficits in strength and balance, home and environmental hazards, impaired vision, unsafe footwear, and improper use of assistive devices. The implementation strategies are developed in a participatory community planning process. These might include, for example, training of professionals and volunteers, improved availability of exercise classes, and education and raising awareness via newspaper, radio, or lectures.

**Results:**

The study starts in September 2010 and ends in December 2013. It is evaluated primarily by process evaluation as well as by telephone survey.

**Conclusions:**

Physical activity as a key message entails multiple positive effects with benefits on a range of geriatric symptoms. The strength of the design is the development of implementation strategies in a participatory community planning. The problems that we anticipate are the dependency on the stakeholders’ willingness to participate, and the difficulty of evaluating population-based programs by hard end points.

## Introduction

### Falls and Fall-Related Injuries

Falls and fall-related injuries pose a major threat to older peoples’ health and well-being, and are associated with an increased morbidity and mortality. Moreover, fall-related morbidity poses a substantial burden on caregivers and families, and the economic burden on health care systems is highly relevant [1]. With the number of older people constantly increasing, fall-related injuries such as fractures will rise dramatically [2]. The development and implementation of strategies to prevent falls and fall-related injuries have therefore been recognized as an urgent public health challenge.

A variety of risk factors for falls have been identified, and a number of interventions have shown to be effective. Among these are, for example, different types of exercise, removal of environmental hazards, and improvement of vision [3]. Recommendations and guidelines for the prevention of falls that advocate identifying high risk persons, screening for modifiable risk factors, and the targeting of appropriate interventions have been developed [4-7]. Efforts by the US Centers for Disease Control and Prevention (CDC) are an example of such a high risk approach. “Stopping Elderly Accidents, Deaths, and Injuries” is a fall prevention tool kit for health care providers developed by the CDC that incorporates risk assessment, treatment of underlying risk factors, and referral to community-based fall prevention programs [8]. However, such case finding is time consuming, and the cost effectiveness of high risk approaches is unclear [9,10].

### Population Strategies

In contrast to high risk approaches, Rose proposed a “population strategy” for common health problems by shifting “the whole distribution of exposure in a favorable direction” [11]. He argued that when risk is widely distributed in a population, small changes in behavior across the whole population are likely to yield greater improvements than large changes in a few people. Therefore, targeting preventive interventions only at high risk individuals would have minimal effect on the population’s health. The risk of falling can be regarded as widely distributed among the general older population, and a substantial proportion of falls occur among people who are neither frail nor identified as being at a high risk of falling [12]. A population shift with regard to fall prevention in this case means, for example, an overall increase of physical activity in the older population, enhanced participation in exercise and fall prevention classes, or reduction of home and environmental hazards. To achieve this, various strategies such as policy development, raising awareness, or influencing of architecture have to be implemented. This approach primarily aims at a shift from preventive to health promoting interventions.

Few population-based programs for fall prevention provide evidence on their effectiveness and information on successful strategies for implementation and motivation [13]. An example of a successful population-based intervention is the Australian “Stay On Your Feet” program. The four year multi-strategic approach addressed fall-related risk factors, knowledge, attitudes, and behaviors targeting home dwelling people 60 years and older. The strategies included, for example, raising awareness, policy development, education, or home hazard reduction. It was delivered via brochures, advertisements, television, and radio; and cooperated closely with local physicians and health care professionals [14,15].

The impact of fall prevention on a population level depends on the participation rates and on the wide availability of fall prevention strategies. There are few data on population-based interventions in the interest of estimating the exact participation rate to achieve an impact on the overall fracture rates. Based on Australian data, Day et al calculated 5440 falls prevented, assuming that 1.9% of the eligible Australian population 70 years and older took up Tai Chi classes [16]. In general, implementation of new activities is a complex process that requires new knowledge, skills, and abilities. These must be taught, learned, and used to change the practice of the involved providers, organizations, and the system in which these new activities are delivered [17]. Consequently, the implementation of effective interventions has to be routinely addressed. A challenge specific to the implementation of fall prevention is the need for coordination and integration of more than just one setting and provider. As an example, fall preventive exercise classes must be tailored to different target groups; and sports clubs, charity and health care organizations, or community services might provide them. Such classes might be instructed by therapists, nurses, exercise instructors, or qualified volunteers at peoples’ homes, sports clubs, gymnasiums, community centers, or nursing homes. Financing for the classes might be provided by the health care system, local or state governments, communities, donations, or by fees. Hence, the implementation of just one single effective activity in a community setting requires profound knowledge of the context in order to address all the relevant institutions and individuals. Even more so, the implementation of a variety of activities requires a multi-strategic approach with a high degree of coordination between settings, providers, and systems [18].

Fall prevention activities require a substantial effort from the older people themselves; therefore, motivation is a key issue. However, older people are reluctant to get involved in fall prevention activities and uptake rates for interventions in the community are typically low [9,19]. Although older community dwelling people consider falls to be an important health issue, they tend to minimize their personal risk [20-22]. While raising awareness can improve knowledge about falls, such activities do not necessarily improve the self-perceived risk of falling [23]. Research on motivational aspects has improved the understanding of older peoples’ views and opinions regarding fall prevention. In line with these findings, a recommendation developed by the Prevention of Falls Network Europe advocates to focus on the dissemination of knowledge that physical activity can improve strength and balance, and to promote the immediate benefits of fall prevention interventions [24].

In Germany, to the best of our knowledge, no population-based program for the prevention of falls in older people has been implemented so far. Therefore, the overall aim of the study presented in this paper is the development, implementation, and evaluation of a population-based intervention for the prevention of falls and fall-related injuries in a medium sized city in Germany. In the design of the study, two main measures are defined: (1) the increase of physical activity, and (2) the reduction of modifiable risk factors for falls using existing structures within the community. The strategies to implement these measures are developed in a participatory community planning. In this paper we describe the background and design of the study.

## Methods

### Context

In Germany, care for the elderly by municipalities implies the coordination of services, policy making, planning, and building of infrastructure such as nursing homes. Self-employed physicians, therapists, and hospitals provide health care. The nursing care is provided by for-profit and nonprofit home care services and nursing homes, which are financed by compulsory long-term care insurances with considerable copayment from the older people themselves. Financing of health services is granted by health insurances and reimbursement of services is highly regulated by federal laws.

To date, there is no national German guideline or policy on fall prevention [25]. Over the last few years fall prevention programs have been implemented in many long-term care facilities, but there have been no similar initiatives targeting community dwelling older people [26]. Numerous institutions, such as the German Red Cross, member organizations of the German Federal Sports Organization, and individual therapists offer fall prevention exercises. In most cases course content is based on scientific evidence, but these programs have not been formally evaluated in controlled trials. The quality of instructors varies considerably, ranging from therapists with special qualifications, to qualified volunteers. The fall prevention activities are not usually covered by health insurance plans. Municipalities or housing associations offer counseling on home modifications. The costs of housing adaptations are partially covered for people in need of care by long-term care insurance. The preventive home modifications depend mainly on the initiative of the older people and their caregivers.

Physical activity in the general older population is promoted and offered by sports clubs and numerous local nonprofit providers, further exercise classes are offered by churches, welfare, and volunteer organizations. Most nonprofit exercise class providers rely on qualified volunteers as instructors. Financing for these classes is based on fees, public funding, and fund raising activities. Increasingly, privately run sports clubs are attractive to older people as well for these classes [27].

### Study Design

The study presented is designed as a population-based multi-strategic intervention. The author Last defines a community-based intervention when “the unit of allocation to receive a preventive regimen is an entire community” [28]. In the approach presented, all noninstitutionalized people 65 years and older are addressed. A multi-disciplinary research team comprising of members with a background in physiotherapy, gerontology, public health, and geriatrics conducts the study. It starts in September 2010 and ends in December 2013. It is financed by the Federal Ministry of Education and Research.

### Intervention Community and Population

The intervention community is the city of Reutlingen, a mid sized city in southern Germany with a population of 112,700 people. About 20.00% (n=22,540) of the inhabitants are 65 years and older. The city was chosen for its proximity to the research center conducting the study (45 km), the medium size (compared to other German cities), and the willingness of local partners to cooperate. Reutlingen is characterized by the combination of an urban city center, and smaller rural districts. There are 103 clubs, various welfare, and volunteers’ organizations, as well as churches that all offer exercise classes. There are 73 general practitioners that are represented by a local body, and 16 for-profit and nonprofit ambulatory care services that provide nursing care. Policy making within the municipality has a strong focus on the prevention of institutionalization in old age, and on optimization of care for community dwelling older people with functional limitations. There is no policy regarding fall prevention. The geriatric unit conducting the study does not provide any services in the intervention community.

### Intervention

The study is designed not to identify and target high risk persons, but to address the general older population as defined. To clarify the terminology of the population-based intervention being presented, “measure” is defined as a certain protective factor (eg, increased physical activity), whereas “strategy” refers to the means by which the measure is promoted (eg, education, improved availability) [29].

The two following main measures were up taken: (1) the increase of physical activity, and (2) the reduction of modifiable risk factors for falls such as deficits in strength and balance, home and environmental hazards, impaired vision, unsafe footwear, and the improper use of assistive devices.

The population-based strategies to intervene on the individual, social, and environmental levels are developed in a participatory community planning. A variety of strategies are to be developed within the course of the study. These might include, for example, the training of professionals and volunteers; education and raising awareness via newspaper, radio, posters, and lectures; or improved availability of exercise classes in the form of the development of a directory. Physical activity and its benefits serve as the key message, whereas fall prevention is secondary to this. The immediate benefits, such as maintenance of independence and well-being, become embedded in the strategies as important messages. Consequently, the study is called “Schritt halten - Aktiv älter werden in Reutlingen” (“Keep Up - Active aging in Reutlingen”).

### Process Structure

**Figure 1:**
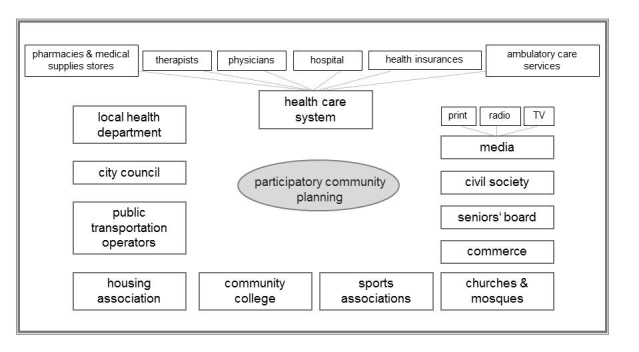
Local stakeholders involved in the participatory community planning.

The study is structured in three stages: (1) Preimplementation period- from July 2010 to September 2011 stakeholders relevant for physical activity, fall prevention, and senior affairs in the community are identified and contacted (Figure 1 shows this organizational structure). The organizational structure of the study is set up. The baseline data is collected from May to July 2011. (2) Implementation period- in September 2011 the development of strategies started and the implementation teams are set up. This period is on going until May 2013. And (3) Follow-up period- after the follow-up data collection from May to July 2013, the financing ends in December 2013. Sustainability poses an important issue of the approach, therefore, the community coalition that is formed is encouraged to further cooperate and meet regularly. For dissemination, the creation of a Web-based portfolio of the developed strategies is planned. It is designed to serve as a template for other municipalities or institutions considering the implementation of fall prevention activities.

### Organizational Structure

Population-based intervention programs are characterized by the shared ownership of the problem and its solution by experts as well as by community members [13]. In this study, we propose a shared decision-making and responsibility for the implementation of strategies. The building of a community coalition for fall prevention is essential at the beginning. In several meetings, the concept of the study is explained and the potential partnerships and contributions from local agencies, organizations, and services are explored. The community coalition is led by a steering committee comprising of high rank representatives of the city’s council, in particular from the senior citizens' office and department of sports, representatives of the geriatric department of the district hospital, the local medical council, the association of sports clubs, the local health department, the building society, the council of senior citizens, the ambulatory care services, and the welfare organizations. The steering committee is built in order to prioritize possible strategies, name appropriate participants of focus groups and implementation teams, and to discuss and solve problems of implementation.

In a second step, focus groups are constituted consisting of local experts, both professionals and volunteers. They discuss and develop portfolios of possible strategies. In dialogue, the steering committee and the researchers rate the proposed strategies. For implementation, teams of local partners are set up with professionals as well as volunteers (so called implementation teams).

The role of the research team is to provide good-practice examples and information for the professionals and volunteers in the field, to set up organizational structures, to coordinate implementation strategies, and to evaluate the processes and outcomes.

### Web-Based Platform of Exercise Classes Example

To illustrate the approach, an example of an already developed and implemented strategy is given, a Web-based platform of exercise classes.

An early conclusion of focus group meetings was the need for creating a directory of existing exercise classes for older people. After a positive vote by the steering committee, an implementation team was set up under the leadership of a researcher. It was decided by the implementation team not only to address older people, but also their caregivers, families, therapists, or exercise instructors with the intention for them to serve as mediators. The implementation team discussed the information need of older people with regards to usability in cooperation with therapists, physicians, and caregivers. The involved stakeholders and associations were the department of sports, association of sports clubs, community college, German Red Cross, senior citizens' office, and the local hiking club. A Web-based solution was chosen over a print version by the team. The pros for the website were that it was modifiable and updateable (eg, adding further information, categories, or new exercise classes), as well as the availability for mediators such as doctors, therapists, or relatives. A con was the fact that a minority of older people are equipped with Internet access. According to that, we assume mediators to be the key to success in order to motivate older inactive persons to exercise. In the future, the website could also serve as a template for an eventual print version. It was decided by the team to offer a detailed description of training methods, information about appropriate target groups, and accessibility of the location, the costs, and the details of contact. An Australian website was chosen as a template, and the same Web service provider was contracted. A questionnaire was sent out to all known providers of exercise classes. When their responses were received, their information was first checked for completeness, and then entered into the Web-based platform by a physiotherapist. The website was presented to and tested with senior citizens, and modified accordingly. The materials for promotion, such as flyers, posters, and newspaper articles were prepared and distributed. The platform [30] started on the Internet in November 2011 with 90 classes, in April 2013 it presents 320 exercise classes. For process evaluation, for example, links to other local websites or page hits are documented.

### Evaluation

The study is evaluated primarily by process evaluation as well as by telephone survey.

### Process Evaluation

To guide other communities in future activities in the field of fall prevention, a process evaluation of strategies implemented in this study is planned. The appropriate evaluation methods have to be developed according to the strategies. Since the development of implementation strategies is part of the on going project, and will be done together with the local stakeholders, the final evaluation methods used cannot yet be presented. Possible methods might be, for example, the uptake of fall prevention activities by the institutions involved or compliance of the target group. To present the results of the evaluation, a website offers interested parties a “construction kit”, from which various measures and strategies for action can be “taken”. The platform shall inform, for example, about aims, procedures, costs, involved stakeholders, experiences, and barriers and catalyzers for implementation.

### Telephone Survey

A control region is identified with the city of Ludwigsburg. The city of Ludwigsburg with 88,600 inhabitants, 18.96% of them 65 years and older (n=16,800), is situated 70 km northwest of the intervention region. The political and socioeconomic structure is comparable to the intervention region.

### Recruitment

At baseline and at follow-up (two years later), we plan to recruit two independent samples of home dwelling people 65 years and older in both the intervention and in the control region. The baseline and follow-up samples are independent. The potential participants are identified with the help of two health insurance companies: (1) a random sample of 1000 members is identified in both cities, and (2) the health insurance company will contact them. Health insurance is mandatory for all employee and pensioners in Germany. The socioeconomic structure of the health insurance members is expected to be similar in both cities due to federal regulation of mandatory health insurances. Together, both health insurances cover approximately 12,500 members 65 years and older in Reutlingen, and 7000 members 65 years and older in Ludwigsburg. Those willing to participate are invited to contact the study team by post or telephone, resulting in a self selected sample. A response rate of 20% is expected. We will not control for multiple identification of members.

### The Questionnaire

The respondents are asked for a written informed consent. The interview is conducted via telephone by a trained interviewer. An interviewer manual will be developed.

Physical activity is measured by the physical activity questionnaire recommended for older people [31,32]. The general self-rated health status is assessed by one item of the Short-Form Health Survey [33] (“In general, would you say your health is excellent, very good, good, fair, or poor?”). The Rivermead Mobility Index is used to measure the mobility of the participants. [34]. To assess changes in behavior, for example, concrete plans to start doing physical exercises within the next weeks/months, Prochaska’s transtheoretic model (the stages of change in the modification of problem behaviors) is used [35]. The participants’ fall rates are measured by the retrospective history of falls during the last year. Concerning the fear of falling, a single item asks, “In general, are you afraid of falling over?” (Possible answers = not at all, a little, quite a bit, very much) [36]. Furthermore, an open question about the knowledge of risk factors for falls was conducted (eg, impaired vision, indoor and outdoor environmental hazards, unsafe footwear, wrong medication). Additional questions in the follow-up questionnaire evaluate the reach of the developed and implemented strategies in the intervention community.

### Statistics

The primary outcome is a change in physical activity between the baseline and the follow-up. The secondary outcome measures are a change in the fear of falling, change in behavior, and knowledge about risk factors. In descriptive analyses, frequencies and means/medians for discrete and continuous variables, respectively, will be calculated. Logistic and linear regression models will be applied to determine differences between the baseline and the follow-up. Adjustment for relevant covariates like age and sex will be performed. Adjusted odds ratios and regression coefficients will be shown with 95% confidence intervals. The Ethics Committee of Ulm University obtained institutional review board approval for the interviews (not for the design and implementation of the overall project).

## Results

The study starts in September 2010 and ends in December 2013. It is evaluated primarily by process evaluation as well as by telephone survey. A detailed description of the process evaluation results will be presented at the Schritt Halten website [37].

## Discussion

### Aims of the Study

The aim of this study is to develop, implement, and evaluate a population-based intervention for the prevention of falls and fall-related injuries using existing structures and resources within the community.

The approach implies physical activity and its benefits as the key message, whereas fall prevention is secondary to this. Knowledge, skills, and attitudes concerning fall and fracture prevention are distributed primarily to health care professionals and exercise instructors. This decision is based on two reasons. First, the self-perceived risk of falling is often judged too optimistically, and older people might regard falls as a relevant problem for others, but not for themselves [20,22,38]. If older people do not believe that they are at risk of falling, they are unlikely to start fall prevention measures. There is a consensus that fall prevention, per se, should not be the headline of health promotion strategies. Rather, the wider benefits of exercise and fall prevention should be the key messages, with a focus on the maintenance of health and independence [22,24,39]. Second, many older people demonstrate more than one geriatric syndrome and more than one chronic condition. Distinct geriatric syndromes, such as falling, share risk factors with other syndromes and chronic conditions, and they contribute to one another [40]. It seems unrealistic to assume that older people are able and willing to adhere to several specific interventions at the same time [41]. Therefore, health promotion activities should define interventions with benefits on several risk categories. Physical activity entails multiple positive effects with benefits on a range of geriatric syndromes, such as loss of independence [42,43], and cognitive decline [44,45]. It offers a generic rather than a disease specific preventive intervention.

### Study Strengths

The strength of the study design is the joint development of implementation strategies in a participatory community planning, involving partners, for example, from the health care system, the community, and older people themselves. The risk of falling shares parallels with other chronic conditions, such as diabetes. In both conditions, the occurrence of an acute event (a cardiovascular event in the case of diabetes, or a fracture in the case of a risk of falling) can be reduced by preventive efforts, like changes in lifestyle by older people themselves, through professionals and providers [18]. In line with the Innovative Care for Chronic Conditions framework of the World Health Organization, population-based fall prevention programs are operating within the structures of a community [13,46].

### Study Limitations

A problem that we anticipate is the dependency on the stakeholders’ willingness to be actively engaged in the project. To maximize cooperation, potential barriers and facilitators for each partner have to be identified at the beginning. A further difficulty of population-based programs is the evaluation of hard end points. Fracture rates, awareness, or lifestyles might not change within the three year duration of the study. These processes, as well as changes of structures and procedures in communities, might take many years and require longer observation periods. During this observation period, other factors, like migration or secular trends, can influence the effects of these community level approaches, which might lead to dilution bias. Therefore, Lindholm and Rosén, for example, state that hard end points are inappropriate options for community-based primary interventions [47]. Rather, our study intents to identify strategies that are feasible and acceptable in the context of the German social and health care system.

## Abbreviations

CDC: US Centers for Disease Control and Prevention
